# Studies of transmembrane peptides by pulse dipolar spectroscopy with semi-rigid TOPP spin labels

**DOI:** 10.1007/s00249-021-01508-6

**Published:** 2021-02-28

**Authors:** Igor Tkach, Ulf Diederichsen, Marina Bennati

**Affiliations:** 1 Max Planck Institute for Biophysical Chemistry, RG Electron-Spin Resonance Spectroscopy, 37077 Göttingen, Germany; 2 Department of Organic and Biomolecular Chemistry, University of Göttingen, 37077 Göttingen, Germany

**Keywords:** DEER, PELDOR, Pulsed ESR, Dipolar spectroscopy, PDS, Spin label, SDSL, α-TOPP, β-TOPP, Transmembrane peptide, β-peptide

## Abstract

Electron paramagnetic resonance (EPR)-based pulsed dipolar spectroscopy measures the dipolar interaction between paramagnetic centers that are separated by distances in the range of about 1.5–10 nm. Its application to transmembrane (TM) peptides in combination with modern spin labelling techniques provides a valuable tool to study peptide-to-lipid interactions at a molecular level, which permits access to key parameters characterizing the structural adaptation of model peptides incorporated in natural membranes. In this mini-review, we summarize our approach for distance and orientation measurements in lipid environment using novel semi-rigid TOPP [4-(3,3,5,5-tetramethyl-2,6-dioxo-4-oxylpiperazin-1-yl)-L-phenylglycine] labels specifically designed for incorporation in TM peptides. TOPP labels can report single peak distance distributions with sub-angstrom resolution, thus offering new capabilities for a variety of TM peptide investigations, such as monitoring of various helix conformations or measuring of tilt angles in membranes.

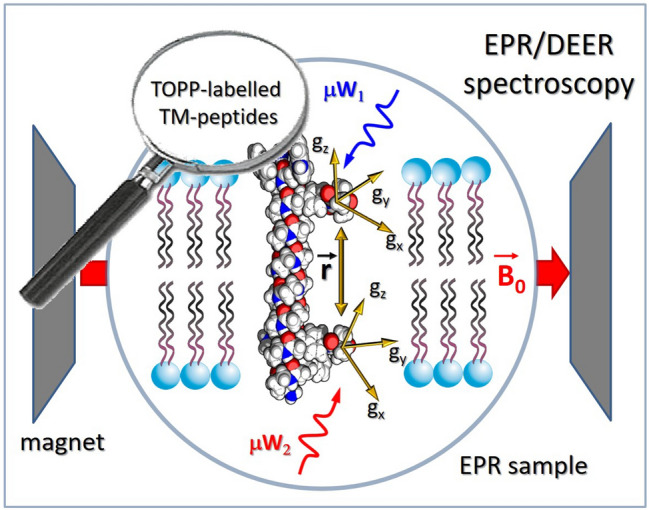

## Introduction

Transmembrane peptides and proteins undertake numerous biological functions in cells and are essential for molecular transport, signaling and membrane fusion (Ghirlanda and Senes 2013; Holt and Killian 2010; Seddon et al. 2004). Many peptides exhibit antibiotic activity (Brogden 2005; Mahlapuu et al. 2016). Investigations of TM peptides in biological membranes require biophysical methods that access their conformational transitions and dynamics (Sun et al. 2015). Even though X-ray is an excellent tool in obtaining molecular structures, preparation of biologically relevant crystals is challenging and the obtained information might not represent the conformational state of interest. Instead, protein folding, oligomerization, and peptide penetration into membranes can be investigated by biophysical methods, which allow for molecular studies in non-crystallized, glass-like media, or even at room temperatures, i.e. at conditions nearly reproducing physiological conditions. For instance, EPR or NMR magnetic resonance techniques are particularly suited as they can detect these species in lipid environments (Bordignon et al. 2019; Ge et al. 1994; Jaipuria et al. 2018; Loura and Prieto 2011; Mayo et al. 2018; Möbius et al. 2013; Sahu et al. 2014; Sani and Separovic 2015; Taylor et al. 2017; Wirmer-Bartoschek and Bartoschek 2012).

Pulse dipolar spectroscopy (PDS) encompasses a class of EPR-based methods, which measures the dipolar interaction between paramagnetic centers on the nanoscale and permits studies of biomolecules close to their physiological conformation (Abdullin and Schiemann 2020; Borbat and Freed 2018; Jeschke 2018; Spindler et al. 2018). The technique is applicable to natural paramagnetic centers or specifically designed spin labels separated by approximately 1.5–10 nm. In combination with site-directed spin labeling (SDSL) (Altenbach et al. 1990; Bordignon 2012), the method can provide important information about structure and conformational dynamics of diamagnetic proteins lacking natural paramagnetic centers.

The most widespread technique, which was initially proposed by (Milov et al. 1984, 1998), is called DEER (double electron–electron resonance) or alternatively PELDOR (pulse electron–electron double resonance). The implementation of the dead-time free, four-pulse variant by Spiess and coworkers (Martin et al. 1998; Pannier et al. 2000), in combination with suited methods for analysis (Chiang et al. 2005; Jeschke et al. 2002, 2006), made a crucial impact for a general establishment of PDS in structural biology. Other EPR techniques for measuring electron–electron dipolar couplings include the “2 + 1” sequence (Astashkin et al. 1998; Kurshev et al. 1989), double-quantum coherence (DQC) EPR (Borbat and Freed 1999, 2018), single-frequency technique for refocusing (SIFTER) dipolar couplings (Jeschke et al. 2000a) and relaxation-induced dipolar modulation enhancement (RIDME) (Kulik et al. 2001; Milikisyants et al. 2009). For distances shorter than 1.5 nm, continuous wave (CW)-EPR or electron-nuclear double resonance (ENDOR) can also be used (Meyer et al. 2020; Rabenstein and Shin 1995; Sahu et al. 2014; Zanker et al. 2005; Zhang et al. 2009). In addition, orientation-related structural parameters can be measured by monitoring either angular dependencies of dipolar spectra or hyperfine coupling (Denysenkov et al. 2006; Dzikovski et al. 2004; Inbaraj et al. 2007; Newstadt et al. 2009).

**Fig. 1:**
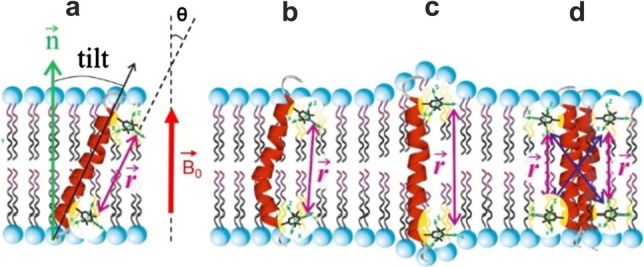
Illustration of possible scenarios of peptide adaptation within a membrane (Holt and Killian 2010) which can be studied using PDS and SDSL. **a** Tilting of the helix, **b** bending of the backbone, **c** stretching of the lipid acyl chains, **d** peptide aggregation

In the studies of TM peptides and proteins, PDS has gained significance (Bordignon et al. 2019; Sahu et al. 2013) as it can provide a reliable measurement of parameters, characterizing peptides embedded into a lipid environment. Indeed, changes of a peptide structure, its folding, bending and specific tilt in the membrane can be monitored either by measuring the distance between specifically attached spin labels or the orientation of the spin–spin dipolar vector with respect to the magnetic field, as illustrated in Fig. 1. Adaptation, which involves membrane stretching, can be assessed by comparing the inter-spin distances in solutions with those in lipids, combined with tilt measurements. Whereas, agglomeration is commonly revealed by analyzing PDS signal strengths and spin–spin relaxations at different label concentrations and media (solutions vs. lipids) (Jeschke 2018).

The quality of PDS data and the accuracy of their interpretation strongly relies on properties of a chosen paramagnetic label. The most established class of EPR spin probes are nitroxide-based labels, most notably the frequently used MTSSL (Altenbach et al. 1990; Fanucci and Cafiso 2006; Hubbell and Altenbach 1994). Through S–S bond formation, the label can be easily attached to cysteines, which either present as the native amino acids or can be introduced in proteins by site-directed mutagenesis. It displays high stability in absence of reducing agents and, due to a small size, the native secondary and tertiary structure of the protein is usually well-retained. However, MTSSL is highly flexible, which leads to complex distance distributions especially in lipid media (Halbmair et al. 2016). Thus, an interpretation of the observed distances delivered by the label is often complicated. To overcome this issue, we introduced two semi-rigid nitroxide labels, i.e. 4-(3,3,5,5-tetramethyl-2,6-dioxo-4-oxylpiperazin-1-yl)-L-phenylglycine and 4-(3,3,5,5-tetramethyl-2,6-dioxo-4-oxylpiperazin-1-yl)-D-β^3^-homophenylglycine, called α-TOPP and β-TOPP, respectively, which enable structural investigation of α- and β-transmembrane peptides in their natural environment (Stoller et al. 2011; Wegner et al. 2019). The labels did not show influence on the peptide secondary structures and reported sharp distance distributions.

This review is organized as follows: the section Theoretical background summarizes the essentials of DEER/PELDOR spectroscopy. In Investigations of TM peptides with TOPP labels, the properties of new semi-rigid α- and β-TOPP labels for distance and orientation measurements on TM peptides are reviewed. Structural information from TOPP orientations covers orientation selective experiments at high magnetic fields and Perspectives: measurements of tilt angles gives some prospects for PDS on membranes.

## Theoretical background

The theory of the PELDOR/DEER experimental has been reviewed in numerous articles. We will summarize here the main equations defining the important parameters detected in the experiment according to the recent review by (Jeschke 2018).

The dipole–dipole interaction between magnetic moments of two electron spins A and B separated by distance *r*_AB_ can be represented by the Hamiltonian:

1 $$\hat{H}_{dd} \left( {r_{AB} } \right) = \hat{S}_{A}^{T} {\varvec{D}}_{{{\varvec{AB}}}} \hat{S}_{B}$$

where $\hat{S}_{A}$ and $\hat{S}_{B}$ are the spin vector operators for spin A and B, respectively, and ${\varvec{D}}_{{{\varvec{AB}}}}$ is the dipolar interaction tensor.

If the dipolar coupling is small compared to the electron Zeeman interaction and the g-tensor values characterizing the Zeeman interaction of each electron are only weakly anisotropic, the dipolar Hamiltonian can be rewritten in a simple form linking the dipolar interaction with the orientation of the interconnecting vector *r*_AB_ towards the magnetic field:

2 $$\hat{H}_{dd} \left( {r_{AB} } \right) = \hbar \cdot \omega_{dd} \left( {1 - 3cos^{2} \theta } \right)\hat{S}_{Z,A} \hat{S}_{Z,B}$$

with $\hat{S}_{Z,A}$ and $\hat{S}_{Z, B}$ representing the z-components of spin vector operators, and $\omega_{dd}$ being a dipolar splitting constant expressed in terms of the effective *g*-values and inter-spin distance *r*_AB_:

3 $$\omega_{dd} = \frac{{\mu_{0} \mu_{B}^{2} }}{4\pi \hbar }\frac{{g_{A} \left( {\Theta ,\Phi } \right)g_{B} \left( {\Theta ,\Phi } \right)}}{{r_{AB}^{3} }}$$

Here, $\Theta$ and $\Phi$ are the azimuthal angles defining the orientation of the *g*-tensor frame towards the magnetic field *B*_0_, $\mu_{B}$ is the Bohr magneton and $\theta$ is the angle of the interconnecting vector *r*_AB_ with respect to *B*_0_.

**Fig. 2:**
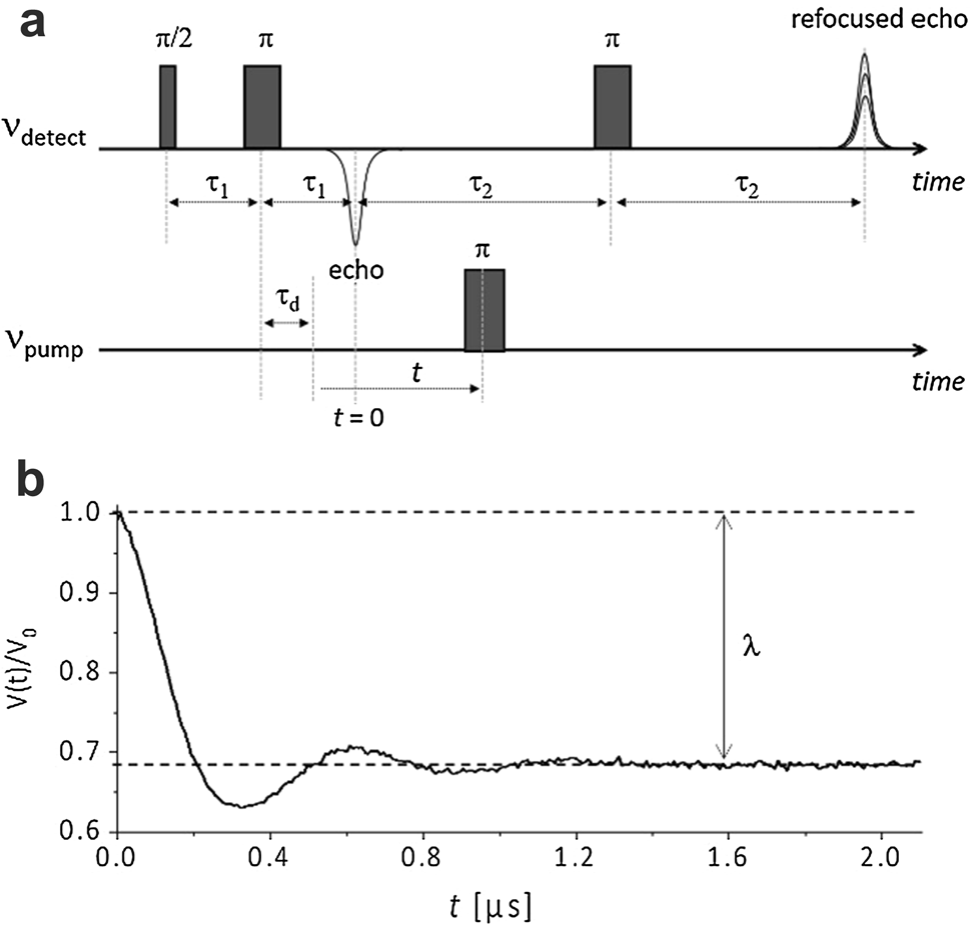
**a** Four-pulse DEER/PELDOR sequence. The pump pulse is shifted between the 2^nd^ and the 3^rd^ detection pulses to induce the modulation of the refocused electron spin echo if dipolar coupling exists. The integrated echo signal is recorded as a function of time *t*. The modulation frequency encodes the information on distances between the electron spins. Times τ_1_ and τ_2_ are delays between the pulses in the mw detection sequence. τ_d_ is a delay for the pumping pulse. **b** A typical normalized DEER/PELDOR trace and its modulation depth λ

The DEER/PELDOR sequence used in our studies is illustrated in Fig. 2a, whereas a typical dipolar time trace is depicted in Fig. 2b. The sequence requires two microwave frequencies to pump and detect each electron spin of the target biradical, respectively. It consists of a refocused echo sequence with pulses at fixed positions for the observer frequency (*ν*_detect_) and a single π-pulse at variable positions for the pumping frequency (*ν*_pump_) (Pannier et al. 2000).

The DEER/PELDOR signal is recorded in the form of the refocused-echo intensity evolution $V\left( t \right)$ (see Fig. 2b) depending on the position *t* of the pump pulse within the time interval $\left( {\tau_{1} + \tau_{2} - \tau_{d} } \right)$. The distance information is encoded in the observed dipolar oscillation and, for nitroxide spin labels, it can be best determined in experiments at EPR frequencies of 9 GHz/0.34 T or 35 GHz/1.2 T. However, since both longitudinal (T_1_) and transverse (T_2_) spin relaxations are critical parameters to observe the echo, the experiment can only be performed at low temperatures, typically at *T* around 30–60 K for nitroxides.

The time evolution of the echo intensity for an ensemble of isolated and randomly oriented spin pairs AB is given by (Pannier et al. 2000):

4 $$V_{{{\text{intra}}}} \left( t \right) = 1 - \int \nolimits_{0}^{\pi /2} \lambda \left( \theta \right) \left[ {1 - {\text{cos}}\left( {\omega_{AB} t} \right)} \right]\sin \theta d\theta$$

where $\theta$ is the angle between the dipolar vector and the external magnetic field, *λ* is the modulation depth parameter as defined by Jeschke (Jeschke 2018). The dipolar frequency $\omega_{dd}$ of an isolated spin pair AB is related to their inter-spin distance *r*_*AB*_ by Eq. 3. For nitroxides at low microwave frequencies, due to broad pulse excitation profiles, *λ* is usually independent on the molecular orientation. Thus, $V_{{{\text{intra}}}} \left( t \right)$ results as a sum of contributions from all possible orientations. In this case, the dipolar spectra exhibit axial symmetry and are represented by so-called Pake patterns (Pake 1948), with singularities corresponding to dipolar frequencies at $\theta$ = 0° and $\theta$ = 90° (refer to illustration of complete and incomplete Pake patterns in Fig. 10a, b). The distance between the two spins can be calculated from the singularity at $\theta$ = 90° using eqs. 2 and 3 solved for *r*_*AB*_*.*

Beside the intramolecular two-spin interaction within a single molecule, inter-molecular interactions between distant electron spins contribute to the signal. Hence, the experimental time trace is expressed as (Jeschke et al. 2000b):

5 $$V\left( t \right) = V_{{{\text{intra}}}} \left( t \right) \cdot V_{{{\text{inter}}}} \left( t \right)$$

For that reason, the recorded time trace is processed to eliminate the inter-molecular contribution $V_{{{\text{inter}}}} \left( t \right)$.

Ideally, the two-spin intra-molecular distance should be extracted by fitting the simulated function $V_{{{\text{intra}}}} \left( t \right)$ in Eq. 4 to the experimental time trace*.* However, the realistic presence of distance distributions renders the inverse problem ill-posed, it means that small statistical variation (noise) of the signal causes strong variation in the solution. Therefore, the data are usually analyzed using Tikhonov regularization (Chiang et al. 2005; Jeschke et al. 2004a), which finds the optimal trade-off between RMSD of the fit and the smoothness of the distance distribution. This procedure was implemented in DeerAnalysis by Jeschke and coworkers (Jeschke et al. 2006), the software used for all our low-field studies. Recently, several methods were reported for treating noisy experimental dipolar spectra and to quantify uncertainties of distance measurements (Edwards and Stoll 2016; Fábregas Ibáñez and Jeschke 2019, 2020; Fábregas Ibáñez et al. 2020; Srivastava and Freed 2017, 2019; Worswick et al. 2018). These methods aim at establishing a good practice of signal post-processing and evaluate the influence of moderate background-fit errors on accuracy of distance determination.

## Investigations of TM peptides with TOPP labels

### TOPP: a novel semi-rigid label for peptide studies

Resolution and sensitivity of EPR-based distance measurements strongly depends on sample preparation conditions and the capability of a spin label to adapt within a biomolecule, which is mainly defined by its inherent flexibility and the length of the linker. In recent studies, it has been shown that distance measurements are largely facilitated in diluted solutions of biomolecules (Dastvan et al. 2010; Halbmair et al. 2016; Stoller et al. 2011; Upadhyay et al. 2008; Wegner et al. 2019). In contrast, for biomolecules embedded in a strongly packed environment, such as peptides or proteins in membranes, inhomogeneous distribution and high local concentrations of spin labels lead to short transverse relaxation times (Dastvan et al. 2010; Jeschke et al. 2004b), which strongly limits the signal-to-noise ratio of dipolar time traces. In addition, the inherent flexibility of the widespread MTSSL label, conjugated to cysteines through formation of a disulfide bond, leads to complex and broadened distance distributions. Depending on the specific goal of the study, a compromise between flexibility and adaptation has to be found.

**Fig. 3:**
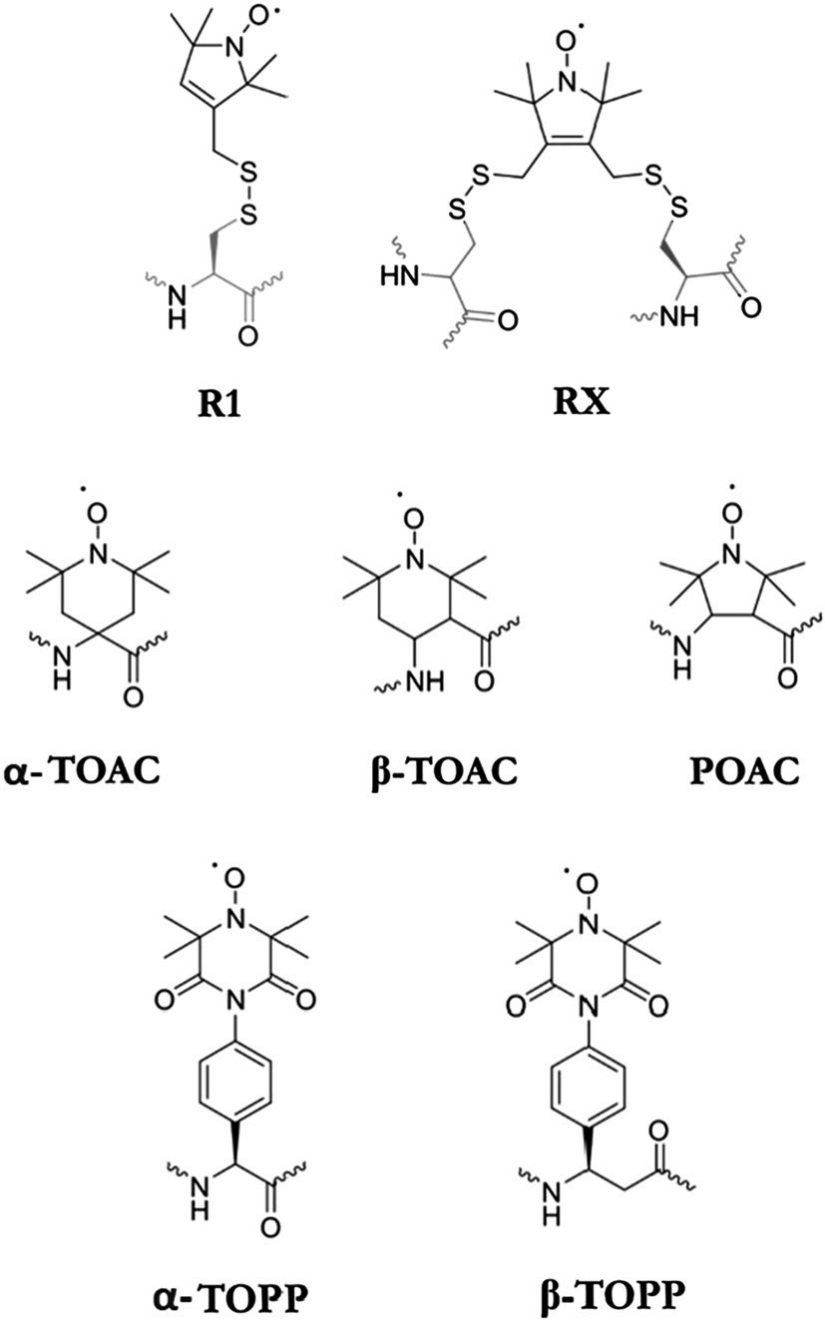
Molecular structures of selected spin labels for protein and peptide labelling. α-TOPP and β-TOPP represent novel semi-rigid labels developed for incorporation into TM peptide by the solid phase peptide synthesis

To restrict label flexibility, nitroxide labels with cross-linked side chain such as the doubly linked RX, the amino acid 2,2,6,6-tetramethylpiperidine-1-oxyl-4-amino-4-carboxyl (TOAC) or 2,2,5,5-tetramethylpyrrolidine-N-oxyl-3-amino-4-carboxylic acid (POAC) have been proposed by (Fleissner et al. 2011), (Marchetto et al. 1993) and (Tominaga et al. 2001), respectively (Fig. 3). RX delivers well-defined distance distributions and is distinctive particularly in context of orientation studies (Norman et al. 2015), but it requires two cysteine residues in close proximity to each other, which limits the range of its applications. In TOAC, the nitroxide group is incorporated in a six-membered ring attached to the backbone α-carbon, which also allows for distance measurements with higher accuracy (Karim et al. 2004). However, TOAC is an achiral amino acid with a tetra-substituted α-carbon affecting the peptide secondary structure (McNulty et al. 2002). Thus, its use remains restricted to cases, in which the amino acid does not perturb the secondary structure of peptides (Gulla et al. 2009; Karim et al. 2004). Similar shortcomings are also valid for the five-membered ring POAC. Furthermore, despite the higher coupling yields compared to TOAC, for POAC the stereoisomers need to be separated before peptide synthesis (Tominaga et al. 2001). Promising is the application of trityl-based spin labels, which allows for distance measurements with a single-frequency dipolar spectroscopy (DQC or SIFTER) at room temperature and in highly reducing cell environment (Fleck et al. 2020; Jassoy et al. 2019; Shevelev et al. 2014). It was also reported that increased sensitivity towards long distances, combined with high stability in cells, can be achieved using Gd^3+^-based labels, particularly if used in measurements at high EPR frequencies (> 90 GHz) (Cohen et al. 2016; Kaminker et al. 2012, 2013; Yagi et al. 2011). However, both trityl-based labels and Gd^3+^ tags are rather large as compared to nitroxides, and therefore, better suited for protein surfaces.

For distance measurements and investigations of peptide secondary structures, including relative orientation of peptide domains, a rigid spin label that can be incorporated into peptides as regular chiral amino acid is desirable. We have introduced the label 4-(3,3,5,5-tetra-methyl-2,6-di-oxo-4-oxylpiperazin-1-yl)-D-phenylglycine (TOPP) (Stoller et al. 2011) as an unnatural amino acid bearing the NO-group with defined orientation in space. The label was synthesized as α- and β-amino acid for the incorporation in α- and β-TM peptides, respectively (Fig. 3). The first comparative analysis of TOPP versus MTSSL using D,L-alternating peptides, indicated a considerable improvement of distance distributions in TFE/EtOH/MeOH (40:40:20) liquid solutions (Stoller et al. 2011). However, it was not successful to insert a TOPP labelled D,L-peptide into a lipid bilayer. Therefore, representative α-WALP (de Planque et al. 1998, 1999) and β-peptides were selected and synthesized to evaluate the capability of α- and β-TOPP to report distances in various lipid environments.

### TOPP vs. MTSSL on WALP24 in solution

**Fig. 4:**
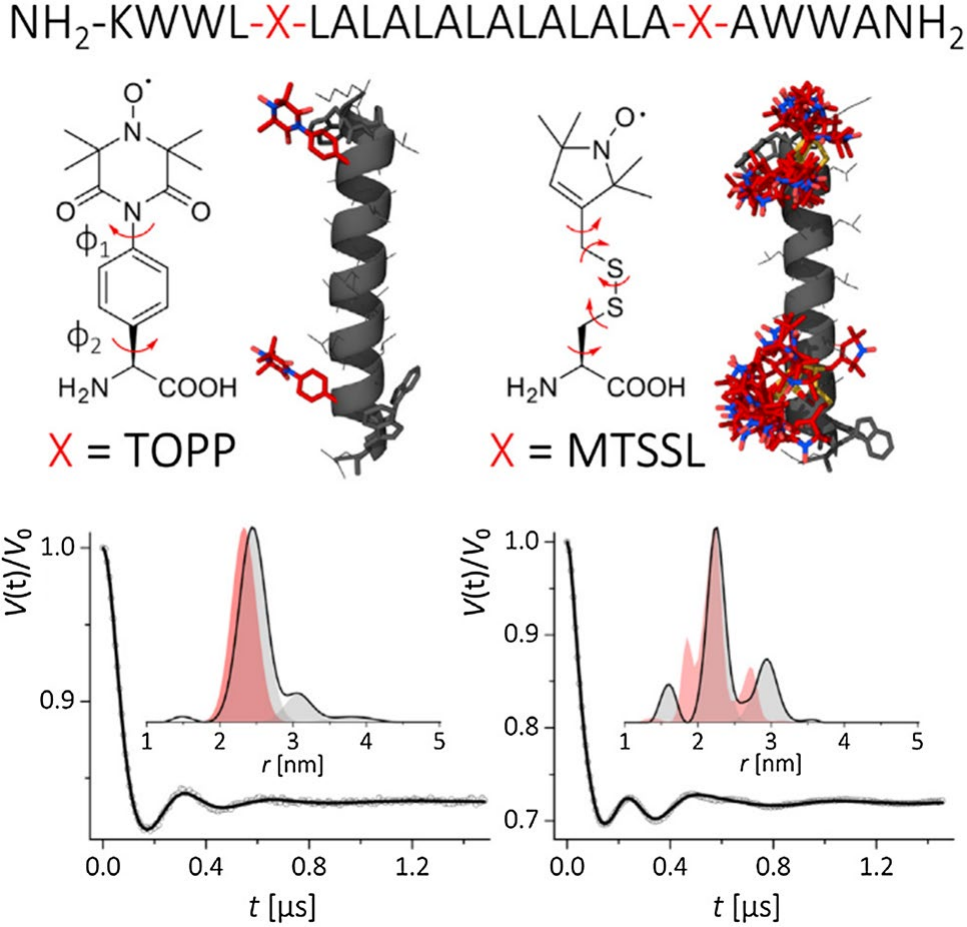
Top: WALP24 peptide sequence. Labelling positions (X) are marked in red. Center: TOPP (left) and MTSSL (right) spin labels and their rotamer distributions as modelled in Ref. (Halbmair et al. 2016). Bottom: Q-band DEER/PELDOR time traces (dots) recorded on TOPP-WALP24 (left) and MTSSL-WALP24 (right) in MeOH and their fits (lines). Experimental and modeled distance distributions are shown in insets (filled grey lines and red areas, respectively). Figure adapted from Ref. (Halbmair et al. 2016)

An initial comparative study of α-TOPP versus MTSSL was performed using WALP24 as a model peptide (Halbmair et al. 2016), which is known to show high structural stability in lipid bilayers (Lueders et al. 2013; Matalon et al. 2013b). Spin-labeled peptides were synthesized by solid-phase synthesis as described in (Halbmair et al. 2016). Figure 4 illustrates typical pulsed dipolar traces at Q-band (34 GHz/1.2 T) from both labeled peptides. Dipolar oscillations are well resolved in both cases. Analysis using Tikhonov regularization (DeerAnalysis) produced a nearly single-peak distance distribution for TOPP in contrast to a broadened distribution, consisting of three well-resolved peak distances, measured with MTSSL.

The observed distance distributions could be explained by simple molecular modelling. For the MTSSL containing peptide, an energy minimization of the peptide structure was performed using PEP-FOLD (Shen et al. 2014) with a cysteine mutation at the label position. Subsequently, possible rotamers of MTSSL, calculated using the MMM software (Polyhach et al. 2011) and taking into account the experimental temperature (50 K), were attached to the optimized backbone. For TOPP-WALP24, an optimized WALP24 peptide structure was computed by inserting a tyrosine at the label position to account for possible aromatic π-interaction. In the geometry optimized structure, the tyrosine was subsequently replaced by TOPP, with the TOPP structure minimized using ab initio and DFT calculations. The predicted peak distance for the TOPP-labeled peptide (average between the O–O, N–O and N–N distances) was found in close agreement with the experiment. In contrast, modeling with MTSSL indicated that rotational flexibility of the single bonds is responsible for the multiple-peak distance distribution (Fig. 4).

Considering that TOPP is a semi-rigid label, attention must be paid as to whether the observed pulse dipolar traces are affected by orientation selection (refer to Structural information from TOPP orientations), which can distort the analysis. The measurements in Fig. 4 were performed on a high-power (170 W) microwave setup with short mw pulses (π < 10–12 ns). Experiments with pump and detection at different resonance positions over the EPR spectrum indicated that there was no dipolar frequency dependence on the detection position, meaning that the conditions for orientation selection could be successfully suppressed.

### TOPP vs. MTSSL on WALP24 in lipids

**Fig. 5:**
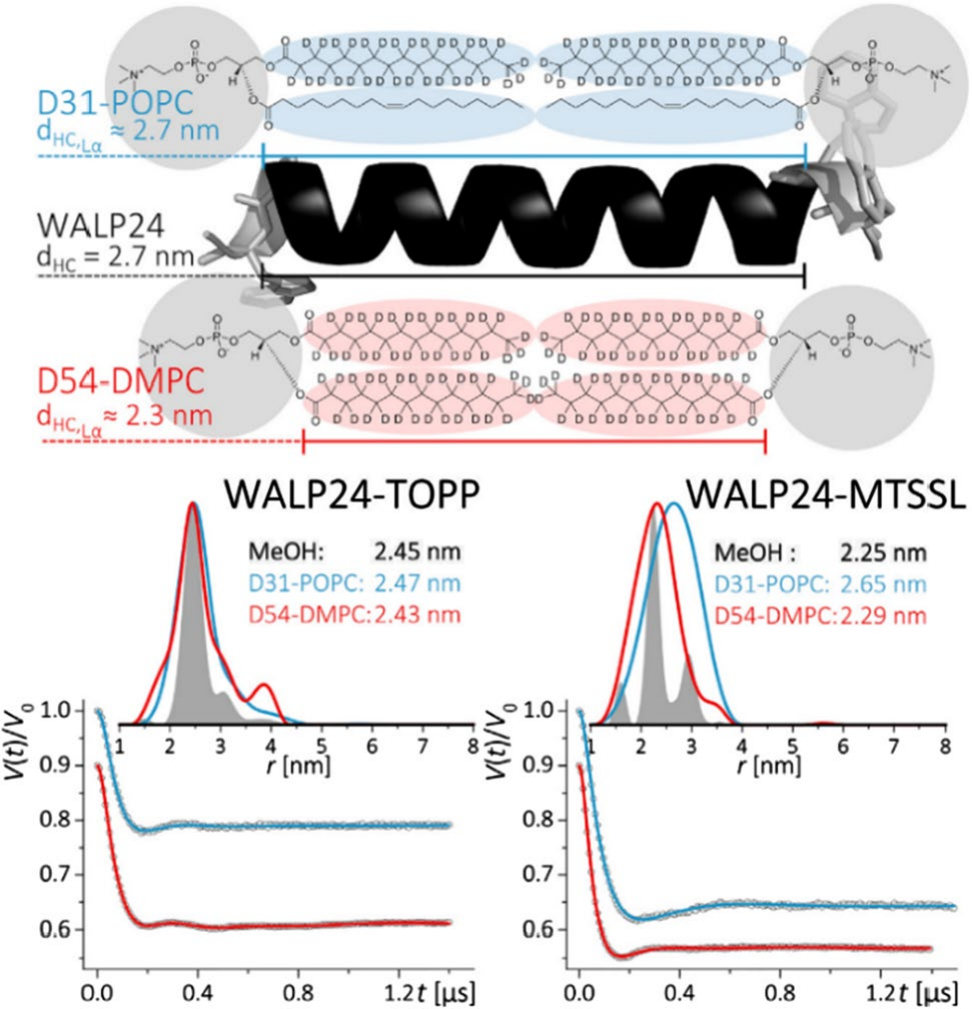
Top: Schematic representation of D31-POPC and D54-DMPC as compared to the length of WALP24. Bottom: Q-band DEER/PELDOR traces of TOPP (left) and MTSSL (right) labelled WALP24 in different environments. Distance distributions obtained using DeerAnalysis are shown in insets. Figure adapted from Ref. (Halbmair et al. 2016)

In the same study, TOPP- and MTSSL-labeled WALP24 peptides were investigated in two different representative lipids, DMPC and POPC (Halbmair et al. 2016). The samples were prepared at specific conditions to form multilamellar lipid vesicles and then rapidly frozen in liquid nitrogen to record DEER (see SI of Halbmair et al. 2016). Rapid freezing was employed to preserve the initial membrane phase and conformational states of peptides. The hydrophobic length of WALP24 does not match the hydrophobic thickness of DMPC (*r* ≈ 2.3 nm, L_α_-phase), however it matches well the hydrophobic thickness of POPC (*r* ≈ 2.7 nm, L_α_-phase) as sketched in Fig. 5, meaning that these two types of lipids are attractive model systems to compare the peptide adaptation under different hydrophobic matching conditions.

To eliminate possible aggregation effects, an optimal peptide/lipid ratio had to be determined. Therefore, a systematic study on WALP24-MTSSL in DMPC was performed with different peptide/lipid ratios ranging from 1:250 up to 1:3000. It was found, that at high and even middle-range peptide/lipid ratios (1:250; 1:1500), *T*_2_ was considerably shortened and the modulation depth was increased comparing to the solution state studies, thus suggesting peptide aggregation. To avoid this, all distance measurements were performed at ratios close to 1:3000. Further sample optimization was achieved using deuterated lipids (D31-POPC, D54-DMPC) permitting to prolong *T*_2_ relaxation.

Figure 5 depicts a comparison of the time traces for TOPP- and MTSSL-WALP24 in DMPC and POPC. The two spin labels account for traces characterized by different distance distributions. Moreover, in lipids, the peak distance between the TOPP labels is nearly the same as in MeOH, i.e. it is not strongly affected by the lipid environment within the experimental error, indicating that the peptide maintains its structure in two different lipids and that the TOPP label equitably reports on the intrinsic peptide conformation. In contrast, utilization of more flexible MTSSL results in broad distance distributions, with mean distances coinciding with the hydrophobic thickness of the lipid bilayer (Fig. 5). It is known that, due to high hydrophobicity, the nitroxide spin labels have a tendency to interact with the interface region between the lipid tails and headgroups (Dzikovski et al. 2012; Matalon et al. 2013a). Thus, the high flexibility of MTSSL allows for adapting to the membrane thickness resulting in loss of information on the internal peptide structure. This observation illustrates that the semi-rigid TOPP-label is better suited for high-resolution distance measurements in lipid environments.

### β-TOPP for structural characterization of β-peptides

β-Peptides belong to another class of model TM peptides attracting considerable attention for studies of interactions with membranes. They are composed of β-amino acids and differ from regular α-peptides by an additional backbone carbon center for each amino acid. The interesting aspect is that, despite the additional CH_2_- group, β-peptides display well-defined secondary structures and rigidity of the helices (Appella et al. 1996; Arvidsson et al. 2001; Brenner and Seebach 2001; Daniels et al. 2007; Seebach et al. 1996); moreover, as side chain substitution can occur either at C_α_ or C_β_, or both, a variety of secondary structures can be created.

Nonetheless, available spin probes for β-peptides, particularly those that can be used for studies in lipid environments, are scarce. β-TOAC was reported as a β spin label with reduced mobility (Schreier et al. 2012; Wright et al. 2005, 2006). Nevertheless, similar to the α-TOAC, the β-analogue is likely to influence the secondary structure (Schreier et al. 2012; Wright et al. 2007). Instead, the β-TOPP amino acid 4-(3,3,5,5-tetramethyl-2,6-dioxo-4-oxylpiperazin-1-yl)-D-β^3^-homophenylglycine (Fig. 3) is suitable for labelling also β-peptides, as was illustrated in Ref. (Wegner et al. 2019). β-TOPP has the advantage that it can be obtained with a high enantiomeric excess and, in contrast to α-TOPP, its incorporation into an oligomer can be achieved without a risk of epimerization.

**Fig. 6:**
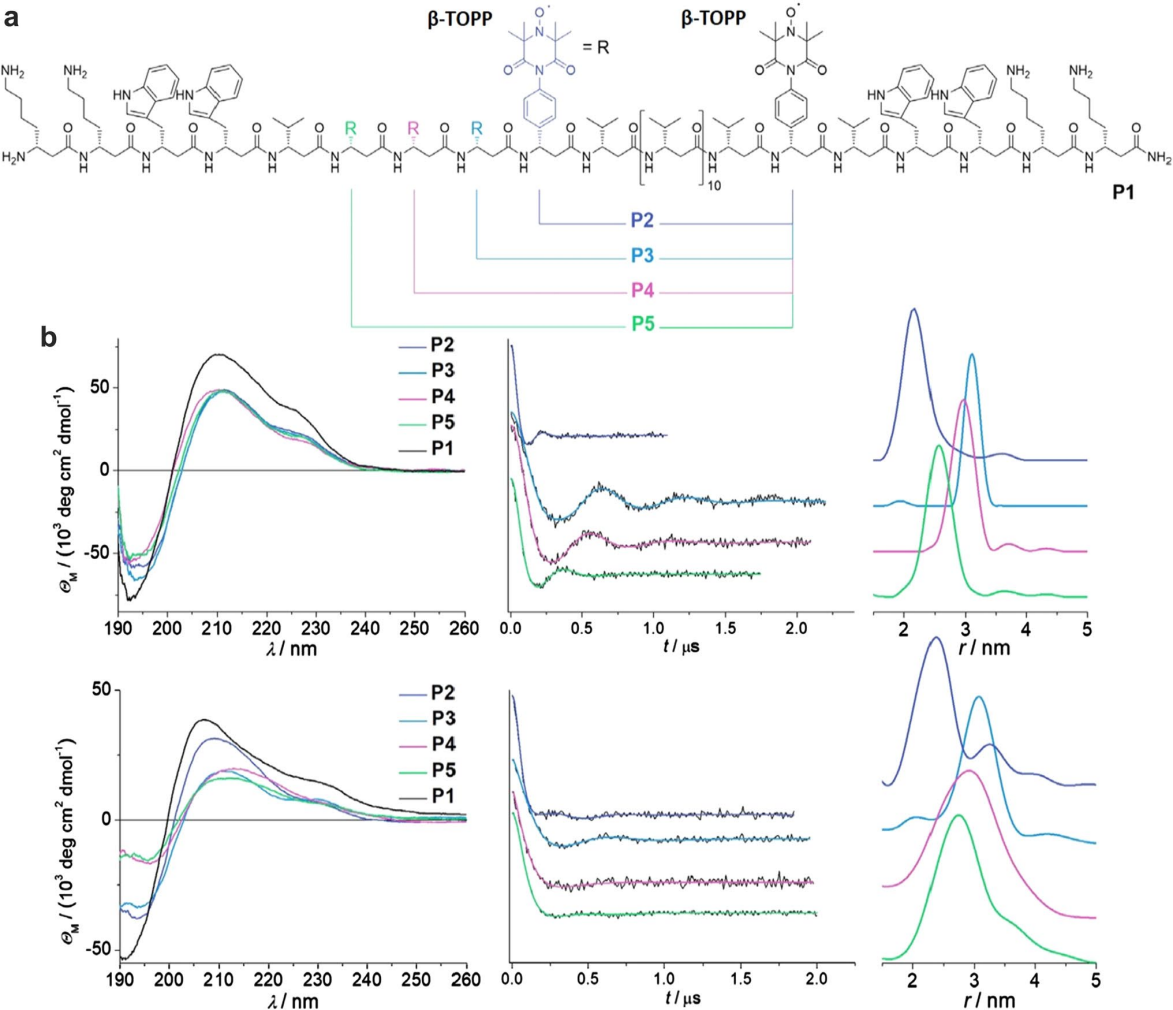
Sequence of the investigated β-peptides and results of CD and DEER spectroscopy. **a** Positions of β-TOPP labels (R) within the peptide sequence for different peptides (P2-P5). **b** Upper row: Measurements in MeOH. Left: CD spectra; Center: Q-band DEER/PELDOR traces (black lines); Right: distance distributions derived with DeerAnalysis. Lower row: Measurements in SUVs of POPC. Left: CD spectra; Center: Q-band DEER/PELDOR traces (black lines) and their DeerAnalysis fits (colored lines); Right: distance distributions derived with DeerAnalysis (colored lines). Colors correspond to peptide color coding in (**a**). Figure adapted from Ref. (Wegner et al. 2019)

EPR experiments were performed to characterize a series of model β-peptides expected to form a 3_14_-helix (i.e. a helix with 3 amino acids forming a turn and 14 atoms in the H-bonded ring of the helix) containing two β-TOPP labels separated by a different number of amino acids (see Fig. 6a). Results were compared in MeOH as well as in multilamellar vesicles (MLVs, POPC and D31-POPC). CW-EPR and electron spin-echo envelope modulation (ESEEM) (Rowan et al. 1965; Van Doorslaer 2018) were used to verify the peptides’ incorporation into the lipid bilayer. Room-temperature CW-EPR spectra in lipids exhibited typical broadening of the nitroxide lines, consistent with an almost uniaxial mobility of the label. For all peptides measured in frozen solutions, time traces showed visible oscillations arising from one predominant distance and well-defined single-peak distance distributions. In the lipid environment, the dipolar oscillations were clearly pronounced as well, though slightly dampened comparing to the solution data.

**Fig. 7:**
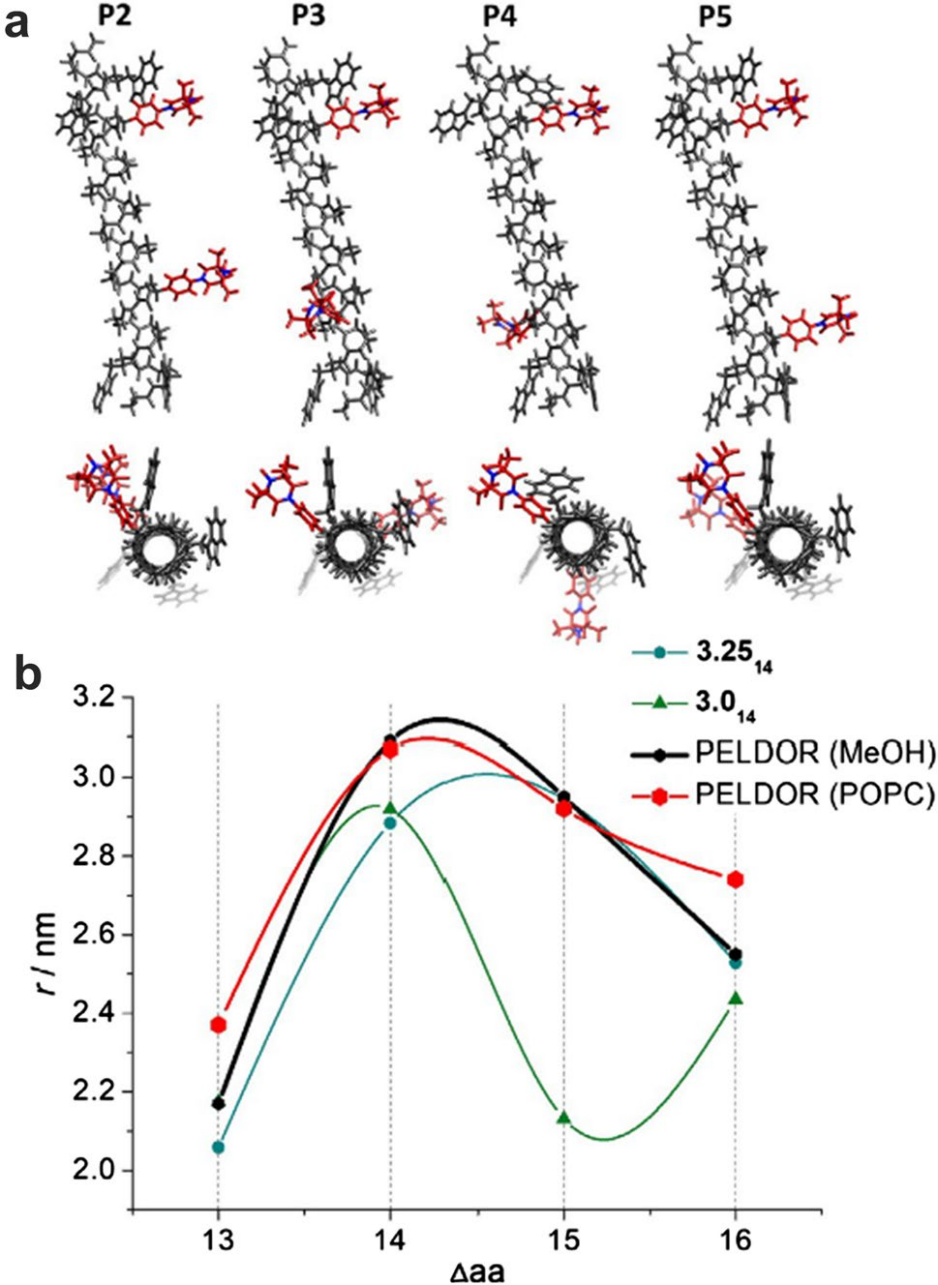
**a** Models of the labelled β^3^-peptides produced according to 3.25_14_ torsion angle set. **b** Inter-spin distances derived with 3.25_14_ (turquoise) and 3.0_14_ (green) backbone angle sets, and experimental peak distances in MeOH (black) and POPC (red) for comparison. The inter-spin distances are plotted against the number of amino acids separating the two spin labels in P2–P5. Figure adapted from Ref. (Wegner et al. 2019)

The well-defined, single-peak distance distributions enabled examination of the helical turn. For that, simplified models of the four doubly-labelled peptides were created and corresponding inter-spin distances were derived (Wegner et al. 2019). Consequently, the inter-spin distances extracted from the models were plotted against the number of amino acids sequentially separating the two labels and compared with those from the experiments (Fig. 7b). The obtained curves indicated a closer agreement of the experimental data with the helix structure specified as 3.25_14_ rather than 3.0_14_, meaning that the helical turn of the β^3^-peptides consists of approximately 3.25 amino acid residues. The study demonstrated that the P2–P5 peptides fold into a 3.25_14_-helix which is conformationally stable in both the MeOH solution and the lipid bilayer.

## Structural information from TOPP orientations

Determination of molecular orientations from spin labels can provide valuable structural information. For instance, the mutual orientation of two spatially rigid or semi-rigid labels can report on orientations of protein or nucleic acid domains, as it was demonstrated in a variety of previous studies (Denysenkov et al. 2008, 2006; Endeward et al. 2009; Marko et al. 2010). This approach can in principle be also employed to report on conformational changes of TM peptides after adaptation in lipids. The investigations require spin labels with well-defined conformations, in which the internal *g*- or hyperfine tensors provide a fixed reference frame within the biomolecule, which can be related unambiguously to the observed dipolar frequencies. While the nitroxide hyperfine tensor has been utilized for orientation measurements at low magnetic fields (Abé et al. 2012; Margraf et al. 2007) and is not field-dependent, the *g*-tensor can be increasingly resolved by raising the polarizing magnetic field and corresponding EPR resonance frequency (Denysenkov et al. 2006).

**Fig. 8:**
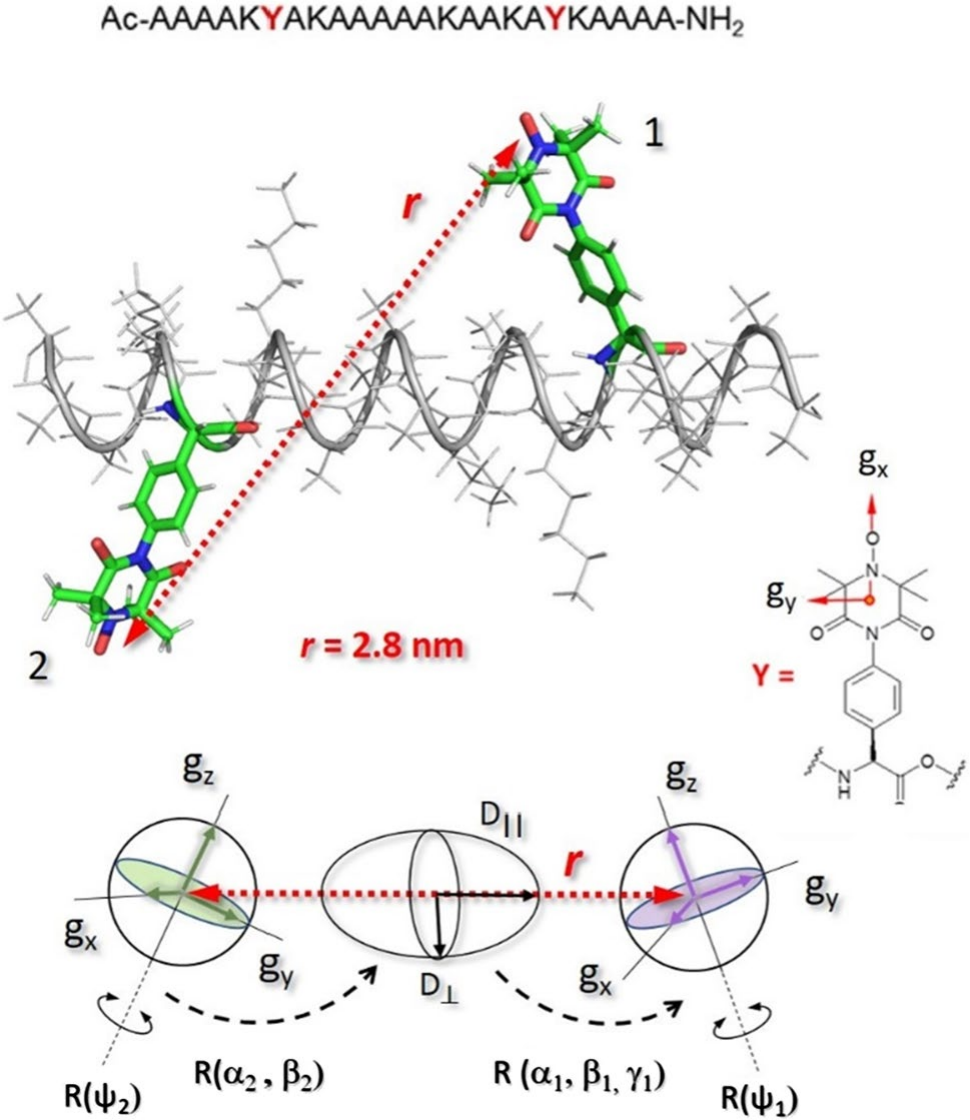
Schematic structure (PyMol, DeLano Scientific LLC) of the α-helical peptide containing α-TOPP-labels (positions Y) employed for orientation studies, adapted from (Tkach et al. 2013). Magnetic *g-*tensors for nitroxides and the tensor transformations defining the mutual label orientation are illustrated. $D_{\parallel }$ and $D_{ \bot }$ represent the parallel and perpendicular principal axis values of the dipolar tensor. $R\left( {\psi_{1} } \right)$ and $R\left( {\psi_{2} } \right)$ describe label librations also considered in the simulations. Inter-spin distance of 2.8 ± 0.2 was determined at 9 GHz

**Fig. 9:**
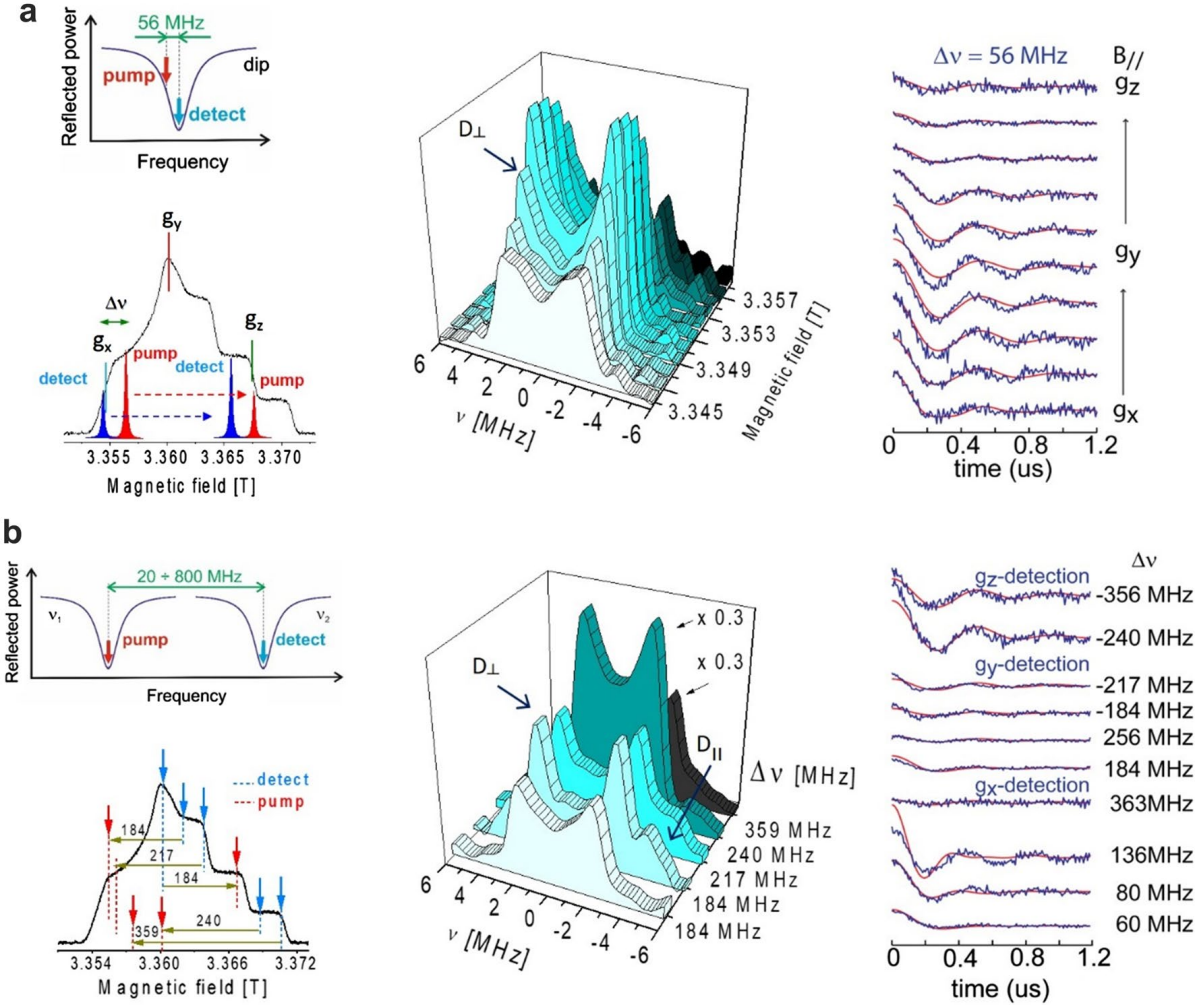
94 GHz DEER/PELDOR of an Ala-rich α-TOPP labelled peptide at fixed **a** and variable **b** frequency separation. **a** Left: schematic tuning picture of a commercial, single mode resonator used for 94 GHz experiments at fixed frequency separation (Δν = 56 MHz) and selected pump and detection position in the EPR line. Center/Right: FT-dipolar spectra (Pake patterns) and time traces obtained with detection across the EPR line. **b** Left: Variable frequency approach with a dual-mode resonator, pump and detection positions are indicated on the EPR spectrum. Center/Right: FT-dipolar spectra and time traces obtained for variable frequency separations and indicated detection positions. Adapted from Tkach et al. (2013)

The capability of determining label orientations with TOPP was examined using an Ala-rich α-peptide (Fig. 8) as reported by (Tkach et al. 2013). As an important prerequisite, quantum chemical calculations predicted that the energy barrier for the internal rotation of α-TOPP around the C–N bond (about 33 kcal/mol) would infer a preferred internal conformation of the label, with the two rings oriented perpendicular to each other. Therefore, all three *g-*tensor principal axes (Fig. 8) were expected to be oriented in space allowing just for a slight distribution only of the *g*_x_ and *g*_y_ axes. The procedure of an orientation measurement consisted in recording dipolar time traces as a function of the microwave excitation or detection resonant positions within the inhomogeneously broadened EPR line (Fig. 9). At 94 GHz/3.4 T, selective mw pulses burn holes in the broad EPR line and excite brunches of molecules according to their effective *g*-values, related to a particular orientation with respect to the magnetic field, *B*_*0*_.

Since the 94 GHz EPR resonances of nitroxides are spread over hundreds of MHz (about 500 MHz at 94 GHz), the feasibility of the double resonance DEER/PELDOR experiment required first the development of a high-frequency microwave resonator that can enhance two microwave frequencies with separation covering the width of the EPR spectrum. For this reason, a novel dual-mode resonator (Tkach and Bennati 2013; Tkach et al. 2011) was designed and implemented, which supported two cylindrical modes and enabled to tune their frequency separation over a broad frequency range, well beyond the nitroxide spectral width. This feature could be utilized later on also for orthogonal nitroxide/Gd^3 + ^distance measurements (Kaminker et al. 2013) as well as for distance measurements with Gd^3 + ^/Gd^3 + ^spin tags (Cohen et al. 2016).

Figure 9 illustrates typical dipolar traces recorded either with a constant pump-detect frequency separation (Δν = 56) MHz and changing the detection field position, or by varying both Δν and the detection field position across the EPR spectrum, which is only possible with the dual-mode resonator (Tkach et al. 2011). Selective pulses, resonant at different positions of the EPR line, excite molecular orientations according to their *g-*values. This selection leads to observation of incomplete powder patterns (Fig. 9, center), from which the mutual orientation of the labels can be reconstructed. As a result, for the fixed frequency separation only one main dipolar frequency component, i.e. corresponding to dipolar vector orientations $\theta$ = 90° in Eq. 2, was observed (marked as $D_{ \bot }$ in Fig. 9a). In contrast, by using the variable Δν, time traces with dipolar frequency components at $\theta$ = 0° could be detected as well ($D_{\parallel }$ in Fig. 9b). The latter frequency provides the strongest restraints to compute the label orientations.

The mutual orientation of the label was extracted by fitting the ensemble of time traces with a set of five Euler angles describing two consecutive rotations $R\left( {\alpha_{1} ,\beta_{1} ,\gamma_{1} } \right) \cdot R\left( {\alpha_{2} ,\beta_{2} } \right)$, defining the orientation of the labels towards the main axis of the dipolar tensor (see Fig. 8). A possible distribution of label orientations along the NO axis was considered allowing for small librations with angles Ψ_1_ and Ψ_2_ (Fig. 8). Examination of residuals around the optimal parameter values indicated that the experimental data recorded with the variable ∆ν pose higher constraints for the solutions, which were well-defined within 10°–15° errors in Euler angles. As a drawback, it was found that a total of 16 symmetry-related combinations of the Euler angles delivered identical fit results, a consequence of the inherent symmetry of the spin Hamiltonian. However, most solutions could be discarded because they were not compatible with the expected molecular structure and only two possible label structures were left for the peptide under study (Tkach et al. 2013).

## Perspectives: measurements of tilt angles

**Fig. 10:**
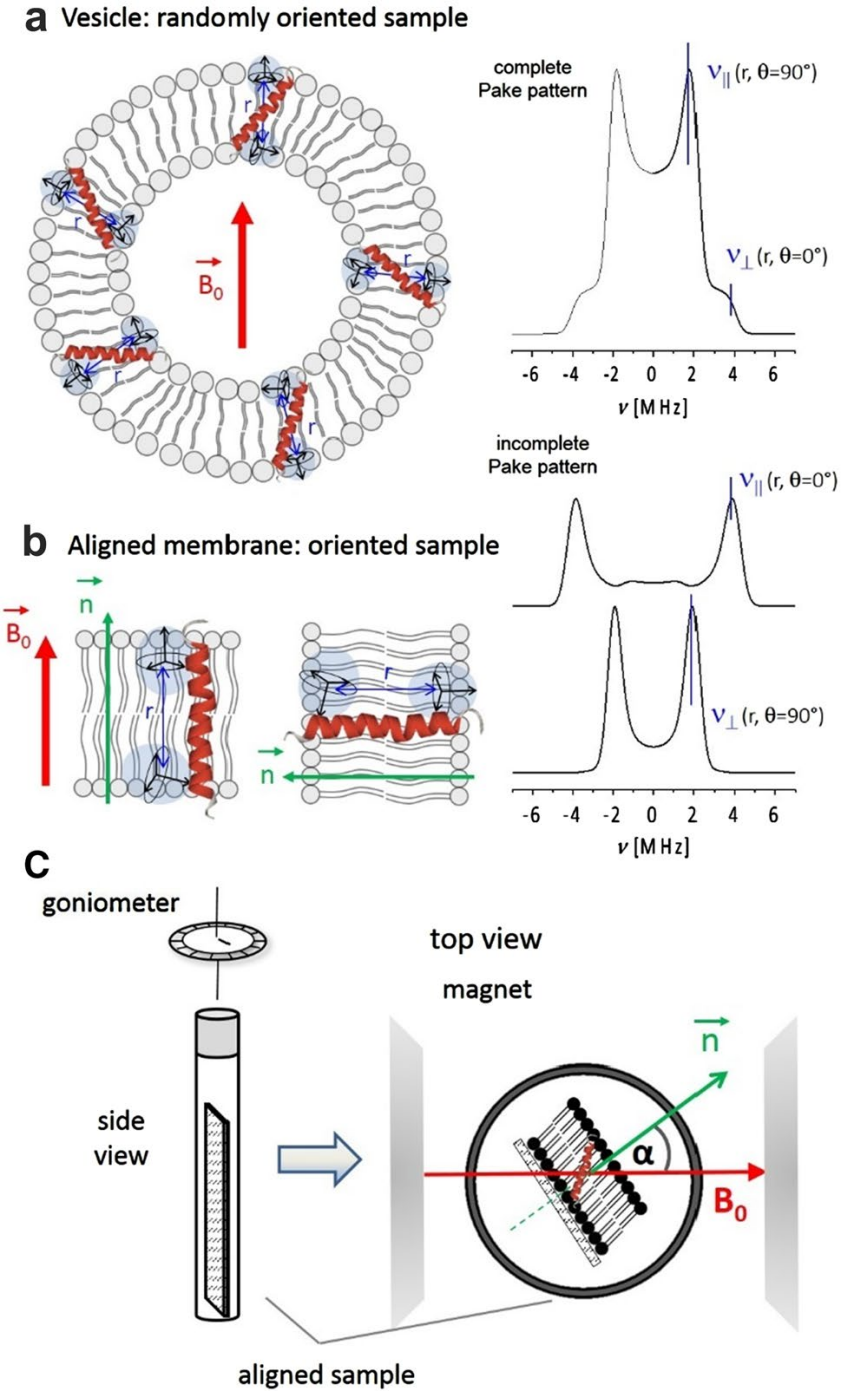
**a**, **b** Examples of randomly and partially oriented peptide samples in membranes and expected complete and incomplete Pake patterns for both arrangements. The incomplete Pake patterns in **B** are representing the arrangements with the interconnecting vectors ***r*** parallel and perpendicular to the magnetic field *B*_*0*_ (upper and lower patterns, respectively). **c** Experimental setup to measure peptide tilt angles in aligned lipids using PDS in combination with SDSL consisting of an aligned membrane on a plate, inserted into an EPR tube. A goniometer can be employed to systematically vary the angle α, from which the dipolar response is detected (Dzikovski et al. 2011)

Peptide adaptation within a lipid bilayer results from hydrophobic mismatch (HMM) when the peptide hydrophobic length does not match the bilayer hydrophobic thickness (Holt and Killian 2010; Killian 1998). One possible adaptation mechanism is the tilt of the peptide within the membrane at a specific angle (see Fig. 1a) (Killian 1998). This angle can be in principle obtained from measurements of dipolar couplings, if the membrane is oriented in the magnetic field, as demonstrated by Freed and coworkers in Dzikovski et al. (2004) and Freed et al. (2010). The situation is schematically illustrated in Fig. 10b, c. The precondition for this experiment is the rigidity of the label along the NO axis, for which the orientation of the dipolar vector remains fixed in space. Accordingly, measurements of dipolar frequencies provide the arrangements of the spin–spin vector towards the magnetic field *B*_0_ and consequently towards the membrane specifically aligned in this field. Labeling positions can be selected such that the dipolar vector is parallel to the peptide axis. The tilt angle can be extracted from simulations of the observed dipolar frequencies as a function of α, the angle between the membrane normal and the direction of the external magnetic field (Fig. 10c). The TOPP label appears very suited for this kind of experiments and work in this direction is in progress to examine its feasibility.

The attractive feature is that these measurements do not require specialized high-frequency EPR instrumentation, however, they rely on a suited sample preparation, i.e. the incorporation of spin-labeled TM peptides in homogenously aligned membranes. Several reports in the past have utilized aligned lipid bilayers combined with ESR and NMR methods and delivered important structural information on different peptides/protein families (Dzikovski et al. 2004, 2011; Inbaraj et al. 2007; Newstadt et al. 2009; Park et al. 2010; Park and Opella 2005). Dzikovski et al. reported studies in various lipid environments on channel and non-channel forms of gramicidin A (GA). Particularly, using the channel form of GA, it was possible to demonstrate how pulse dipolar ESR can be applied to determine the orientation of the membrane-traversing molecule relative to the membrane normal. This information was crucial to study subtle effects of the lipid environment on the gramicidin channel formation (Dzikovski et al. 2004, 2011). Newstadt et al. used CW EPR to determine tilt angles of TOAC-labelled α-helical M2δ domain of the nicotinic acetylcholine receptor (AChR) in aligned membrane media (Newstadt et al. 2009). As another type of aligned lipid media mimicking biological membranes, oriented bicells can also be used for orientation selective measurements (McCaffrey et al. 2015; Sanders and Prosser 1998). All these studies indicate that protocols for preparation of aligned membranes are available. Such protocols in combination with the introduction of semi-rigid spin labels in TM peptides or proteins as well as the development of increasingly sophisticated pulse EPR methods will provide a versitile playground for future investigations of this important class of biomolecules.

## References

[CR1] Abdullin D, Schiemann O (2020). Pulsed dipolar EPR spectroscopy and metal ions: methodology and biological applications. Chem Plus Chem.

[CR2] Abé C, Klose D, Dietrich F, Ziegler WH, Polyhach Y, Jeschke G, Steinhoff H-J (2012). Orientation selective DEER measurements on vinculin tail at X-band frequencies reveal spin label orientations. J Magn Reson.

[CR3] Altenbach C, Marti T, Khorana HG, Hubbell WL (1990). Transmembrane protein-structure: spin labeling of bacteriorhodopsin mutants. Science.

[CR4] Appella DH, Christianson LA, Karle IL, Powell DR, Gellman SH (1996). β-Peptide foldamers: robust helix formation in a new family of β-amino acid oligomers. J Am Chem Soc.

[CR5] Arvidsson PI, Rueping M, Seebach D (2001) Design, machine synthesis, and NMR-solution structure of a β-heptapeptide forming a salt-bridge stabilised 3-helix in methanol and in water. Chem Commun 649–650. 10.1039/B101085I

[CR6] Astashkin AV, Hara H, Kawamori A (1998). The pulsed electron-electron double resonance and "2+1" electron spin echo study of the oriented oxygen-evolving and Mn-depleted preparations of photosystem II. J Chem Phys.

[CR7] Borbat PP, Freed JH (1999). Multiple-quantum ESR and distance measurements. Chem Phys Lett.

[CR8] Borbat PP, Freed JH (2018) Dipolar Spectroscopy – Single-Resonance Methods. In: Goldfarb D, Stoll S (eds) EPR Spectroscopy: Fundamentals and Methods. eMagRes Wiley and Sons 465–494. 10.1002/9780470034590.emrstm1519

[CR9] Bordignon E (2012) Site-Directed Spin Labeling of Membrane Proteins. In: Drescher M, Jeschke G (eds) EPR Spectroscopy: Applications in Chemistry and Biology. Springer Berlin Heidelberg, Berlin, Heidelberg, 121–157. 10.1007/128_2011_243 10.1007/128_2011_243 21898205

[CR10] Bordignon E, Kucher S, Polyhach Y (2019) EPR Techniques to Probe Insertion and Conformation of Spin-Labeled Proteins in Lipid Bilayers. In: Kleinschmidt JH (ed) Lipid-Protein Interactions: Methods and Protocols. Springer New York, New York, NY, 493–528. 10.1007/978-1-4939-9512-7_21 10.1007/978-1-4939-9512-7_21 31218631

[CR11] Brenner M, Seebach D (2001). Design, synthesis, NMR-solution and X-Ray crystal structure of N-Acyl-γ-dipeptide amides that form a βII′-type turn. Helv Chim Acta.

[CR12] Brogden KA (2005). Antimicrobial peptides: pore formers or metabolic inhibitors in bacteria?. Nat Rev Microbiol.

[CR13] Chiang YW, Borbat PP, Freed JH (2005). The determination of pair distance distributions by pulsed ESR using Tikhonov regularization. J Magn Reson.

[CR14] Cohen MR, Frydman V, Milko P, Iron MA, Abdelkader EH, Lee MD, Swarbrick JD, Raitsimring A, Otting G, Graham B, Feintuch A, Goldfarb D (2016). Overcoming artificial broadening in Gd^3+^–Gd^3+^ distance distributions arising from dipolar pseudo-secular terms in DEER experiments. Phys Chem Chem Phys.

[CR15] Daniels DS, Petersson EJ, Qiu JX, Schepartz A (2007). High-resolution structure of a β-peptide bundle. J Am Chem Soc.

[CR16] Dastvan R, Bode BE, Karuppiah MPR, Marko A, Lyubenova S, Schwalbe H, Prisner TF (2010). Optimization of transversal relaxation of nitroxides for pulsed electron−electron double resonance spectroscopy in phospholipid membranes. J Phys Chem B.

[CR17] de Planque MRR, Greathouse DV, Koeppe RE, Schafer H, Marsh D, Killian JA (1998). Influence of lipid/peptide hydrophobic mismatch on the thickness of diacylphosphatidylcholine bilayers. A H-2 NMR and ESR study using designed transmembrane alpha-helical peptides and gramicidin. Biochemistry.

[CR18] de Planque MRR, Kruijtzer JAW, Liskamp RMJ, Marsh D, Greathouse DV, Koeppe RE, de Kruijff B, Killian JA (1999). Different membrane anchoring positions of tryptophan and lysine in synthetic transmembrane α-helical peptides. J Biol Chem.

[CR19] Denysenkov VP, Prisner TF, Stubbe J, Bennati M (2006). High-field pulsed electron–electron double resonance spectroscopy to determine the orientation of the tyrosyl radicals in ribonucleotide reductase. Proc Nat Acad Sci.

[CR20] Denysenkov VP, Biglino D, Lubitz W, Prisner TF, Bennati M (2008). Structure of the tyrosyl biradical in mouse R2 ribonucleotide reductase from high-field PELDOR. Angew Chem Int Edit.

[CR21] Dzikovski BG, Borbat PP, Freed JH (2004). Spin-labeled gramicidin A: channel formation and dissociation. Biophys J.

[CR22] Dzikovski BG, Borbat PP, Freed JH (2011). Channel and Nonchannel Forms of Spin-Labeled Gramicidin in Membranes and Their Equilibria. J Phys Chem B.

[CR23] Dzikovski B, Tipikin D, Freed J (2012). Conformational distributions and hydrogen bonding in gel and frozen lipid bilayers: a high frequency spin-label ESR study. J Phys Chem B.

[CR24] Edwards TH, Stoll S (2016). A Bayesian approach to quantifying uncertainty from experimental noise in DEER spectroscopy. J Magn Reson.

[CR25] Endeward B, Butterwick JA, MacKinnon R, Prisner TF (2009). Pulsed electron-electron double-resonance determination of spin-label distances and orientations on the tetrameric potassium ion channel KcsA. J Am Chem Soc.

[CR26] Fábregas Ibáñez L, Jeschke G (2019). General regularization framework for DEER spectroscopy. J Magn Reson.

[CR27] Fábregas Ibáñez L, Jeschke G (2020). Optimal background treatment in dipolar spectroscopy. Phys Chem Chem Phys.

[CR28] Fábregas Ibáñez L, Jeschke G, Stoll S (2020). DeerLab: a comprehensive toolbox for analyzing dipolar EPR spectroscopy data. Magn Reson Discuss.

[CR29] Fanucci GE, Cafiso DS (2006). Recent advances and applications of site-directed spin labeling. Curr Opin Struct Biol.

[CR30] Fleck N, Heubach CA, Hett T, Haege FR, Bawol PP, Baltruschat H, Schiemann O (2020). SLIM: a short-linked, highly redox-stable trityl label for high-sensitivity in-cell EPR distance measurements. Angew Chem Int Ed.

[CR31] Fleissner MR, Bridges MD, Brooks EK, Cascio D, Kálai T, Hideg K, Hubbell WL (2011). Structure and dynamics of a conformationally constrained nitroxide side chain and applications in EPR spectroscopy. Proc Natl Acad Sci.

[CR32] Freed JH, Dzikovski BG, Borbat PP (2010). Channel and nonchannel forms of spin-labeled gramicidin in membranes and their equilibria. J Phys Chem B.

[CR33] Ge MT, Budil DE, Freed JH (1994). Esr studies Ed spin-labeled membranes aligned by isopotential spin-dry ultracentrifugation - lipid-protein interactions. Biophys J.

[CR34] Ghirlanda G, Senes A (2013). Membrane proteins: folding, association, and design. Methods Mol Biol.

[CR35] Gulla SV, Sharma G, Borbat P, Freed JH, Ghimire H, Benedikt MR, Holt NL, Lorigan GA, Rege K, Mavroidis C, Budil DE (2009). Molecular-scale force measurement in a coiled-coil peptide dimer by electron spin resonance. J Am Chem Soc.

[CR36] Halbmair K, Wegner J, Diederichsen U, Bennati M (2016). Pulse EPR measurements of intramolecular distances in a TOPP-labeled transmembrane peptide in lipids. Biophys J.

[CR37] Holt A, Killian JA (2010). Orientation and dynamics of transmembrane peptides: the power of simple models. Eur Biophys J EBJ.

[CR38] Hubbell WL, Altenbach C (1994). Investigation of structure and dynamics in membrane-proteins using site-directed spin-labeling. Curr Opin Struct Biol.

[CR39] Inbaraj JJ, Laryukhin M, Lorigan GA (2007). Determining the helical tilt angle of a transmembrane helix in mechanically aligned lipid bilayers using EPR spectroscopy. J Am Chem Soc.

[CR40] Jaipuria G, Ukmar-Godec T, Zweckstetter M (2018). Challenges and approaches to understand cholesterol-binding impact on membrane protein function: an NMR view. Cell Mol Life Sci.

[CR41] Jassoy JJ, Heubach CA, Hett T, Bernhard F, Haege FR, Hagelueken G, Schiemann O (2019). Site Selective and Efficient Spin Labeling of Proteins with a Maleimide-Functionalized Trityl Radical for Pulsed Dipolar EPR Spectroscopy. Molecules.

[CR42] Jeschke G (2018) Dipolar Spectroscopy – Double-Resonance Methods. In: Goldfarb D, Stoll S (eds) EPR Spectroscopy: fundamentals and methods. eMagRes Wiley and Sons, 1459–1476 10.1002/9780470034590.emrstm1518

[CR43] Jeschke G, Pannier M, Godt A, Spiess HW (2000). Dipolar spectroscopy and spin alignment in electron paramagnetic resonance. Chem Phys Lett.

[CR44] Jeschke G, Pannier M, Spiess HW (2000b) Double electron-electron resonance. In: Berliner LJ, Eaton GR, Eaton SS (eds) Distance measurements in biological systems by EPR. Springer US, Boston, MA, 493–512. 10.1007/0-306-47109-4_11

[CR45] Jeschke G, Koch A, Jonas U, Godt A (2002). Direct conversion of EPR dipolar time evolution data to distance distributions. J Magn Reson.

[CR46] Jeschke G, Panek G, Godt A, Bender A, Paulsen H (2004). Data analysis procedures for pulse ELDOR measurements of broad distance distributions. Appl Magn Resonan.

[CR47] Jeschke G, Wegener C, Nietschke M, Jung H, Steinhoff HJ (2004b) Interresidual Distance Determination by Four-Pulse Double Electron-Electron Resonance in an Integral Membrane Protein: The Na+/Proline Transporter PutP of Escherichia coli. Biophys J 86:2551–2557. https://www.ncbi.nlm.nih.gov/pubmed/15041691 10.1016/S0006-3495(04)74310-6 PMC1304102 15041691

[CR48] Jeschke G, Chechik V, Ionita P, Godt A, Zimmermann H, Banham J, Timmel CR, Hilger D, Jung H (2006). DeerAnalysis2006—a comprehensive software package for analyzing pulsed ELDOR data. Appl Magn Reson.

[CR49] Kaminker I, Yagi H, Huber T, Feintuch A, Otting G, Goldfarb D (2012). Spectroscopic selection of distance measurements in a protein dimer with mixed nitroxide and Gd3+ spin labels. Phys Chem Chem Phys.

[CR51] Kaminker I, Tkach I, Manukovsky N, Huber Th, Yagi H, Otting G, Bennati M, Goldfarb D (2013) W-band orientation selective DEER measurements on a Gd3+/nitroxide mixed-labeled protein dimer with a dual mode cavity. J Magn Reson 227:66–71. 10.1016/j.jmr.2012.11.028 10.1016/j.jmr.2012.11.028 23314001

[CR52] Karim CB, Kirby TL, Zhang ZW, Nesmelov Y, Thomas DD (2004). Phospholamban structural dynamics in lipid bilayers probed by a spin label rigidly coupled to the peptide backbone. Proc Natl Acad Sci USA.

[CR53] Killian JA (1998) Hydrophobic mismatch between proteins and lipids in membranes. Biochimica et Biophysica Acta (BBA) - Reviews on Biomembranes 1376:401–416. 10.1016/S0304-4157(98)00017-3 10.1016/s0304-4157(98)00017-3 9805000

[CR54] Kulik LV, Dzuba SA, Grigoryev IA, Tsvetkov YD (2001). Electron dipole–dipole interaction in ESEEM of nitroxide biradicals. Chem Phys Lett.

[CR55] Kurshev VV, Raitsimring AM, Tsvetkov YD (1989). Selection of dipolar interaction by the “2 + 1” pulse train ESE. J Magn Reson.

[CR56] Loura L, Prieto M (2011). FRET in membrane biophysics. Overv Front Physiol.

[CR57] Lueders P, Jager H, Hemminga MA, Jeschke G, Yulikov M (2013). Distance measurements on orthogonally spin-labeled membrane spanning WALP23 polypeptides. J Phys Chem B.

[CR58] Mahlapuu M, Håkansson J, Ringstad L, Björn C (2016). Antimicrobial peptides. Emerg Categ Therap Agents Front Cell Infect Microbiol.

[CR59] Marchetto R, Schreier S, Nakaie CR (1993). A novel spin-labeled amino-acid derivative for use in peptide-synthesis: (9-Fluorenylmethyloxycarbonyl)-2,2,6,6-Tetramethylpiperidine-N-Oxyl-4-Amino-4-Carboxylic Acid. J Am Chem Soc.

[CR60] Margraf D, Bode BE, Marko A, Schiemann O, Prisner TF (2007). Conformational flexibility of nitroxide biradicals determined by X-band PELDOR experiments. Mol Phys.

[CR61] Marko A, Margraf D, Cekan P, Sigurdsson ST, Schiemann O, Prisner TF (2010). Analytical method to determine the orientation of rigid spin labels in DNA. Phys Rev E Stat Nonlin Soft Matter Phys.

[CR62] Martin RE, Pannier M, Diederich F, Gramlich V, Hubric HM, Spiess HW (1998). Determination of end-to-end distances in a series of TEMPO diradicals of up to 2_8 nm length with a new four-pulse double electron–electron resonance experiment. Angewandte Chem Int Ed.

[CR63] Matalon E, Huber Th, Hagelueken G, Graham B, Frydman V, Feintuch A, Otting G, Goldfarb D (2013a) Gadolinium(III) spin labels for high-sensitivity distance measurements in transmembrane helices. Angew Chem Int Edit Int Edit 52:11831–11834. 10.1002/anie.201305574 10.1002/anie.201305574 24106050

[CR64] Matalon E, Kaminker I, Zimmermann H, Eisenstein M, Shai Y, Goldfarb D (2013). Topology of the trans-membrane peptide WALP23 in model membranes under negative mismatch conditions. J Phys Chem B.

[CR65] Mayo DJ, Sahu ID, Lorigan GA (2018). Assessing topology and surface orientation of an antimicrobial peptide magainin 2 using mechanically aligned bilayers and electron paramagnetic resonance spectroscopy. Chem Phys Lipids.

[CR66] McCaffrey JE, James ZM, Thomas DD (2015). Optimization of bicelle lipid composition and temperature for EPR spectroscopy of aligned membranes. J Magn Reson.

[CR67] McNulty JC, Thompson DA, Carrasco MR, Millhauser GL (2002). Dap-SL: a new site-directed nitroxide spin labeling approach for determining structure and motions in synthesized peptides and proteins. Febs Lett.

[CR68] Meyer A, Dechert S, Dey S, Hobartner C, Bennati M (2020) Measurement of Angstrom to Nanometer Molecular Distances with F-19 Nuclear Spins by EPR/ENDOR Spectroscopy. Angew Chem Int Edit 59:373–379 10.1002/anie.201908584 PMC6973229 31539187

[CR69] Milikisyants S, Scarpelli F, Finiguerra MG, Ubbink M, Huber M (2009). A pulsed EPR method to determine distances between paramagnetic centers with strong spectral anisotropy and radicals: the dead-time free RIDME sequence. J Magn Reson.

[CR70] Milov AD, Ponomarev AB, Tsvetkov YD (1984). Electron-electron double resonance in electron spin echo: Model biradical systems and the sensitized photolysis of decalin. Chem Phys Lett.

[CR71] Milov AD, Maryasov AG, Tsvetkov YD (1998). Pulsed electron double resonance (PELDOR) and its applications in free-radicals research. Appl Magn Reson.

[CR72] Möbius K, Lubitz W, Savitsky A (2013) High-field EPR on membrane proteins: crossing the gap to NMR. Prog Nucl Mag Reson Spectrosc 75:1–49 10.1016/j.pnmrs.2013.07.002 10.1016/j.pnmrs.2013.07.002 24160760

[CR73] Newstadt JP, Mayo DJ, Inbaraj JJ, Subbaraman N, Lorigan GA (2009). Determining the helical tilt of membrane peptides using electron paramagnetic resonance spectroscopy. J Magn Reson.

[CR74] Norman D, Stevens M, McKay J, Robinson J, El Mkami H, Smith G (2015) The use of the Rx spin label in orientation measurement on proteins, by EPR. Phys Chem Chem Phys 18:5799–5806. 10.1039/c5cp04753f 10.1039/c5cp04753f PMC4756314 26426572

[CR75] Pake GE (1948). Nuclear resonance absorption in hydrated crystals: fine structure of the proton line. J Chem Phys.

[CR76] Pannier M, Veit S, Godt A, Jeschke G, Spiess HW (2000). Dead-time free measurement of dipole-dipole interactions between electron spins. J Magn Reson.

[CR77] Park SH, Opella SJ (2005). Tilt angle of a trans-membrane helix is determined by hydrophobic mismatch. J Mol Biol.

[CR78] Park SH, Das BB, De Angelis AA, Scrima M, Opella SJ (2010). Mechanically, magnetically, and "rotationally aligned" membrane proteins in phospholipid bilayers give equivalent angular constraints for NMR structure determination. J Phys Chem B.

[CR79] Polyhach Y, Bordignon E, Jeschke G (2011). Rotamer libraries of spin labelled cysteines for protein studies. Phys Chem Chem Phys.

[CR80] Rabenstein MD, Shin YK (1995). Determination of the Distance between 2 Spin Labels Attached to a Macromolecule. Proc Natl Acad Sci USA.

[CR81] Rowan LG, Hahn EL, Mims WB (1965). Electron-spin-echo envelope modulation. Phys Rev.

[CR82] Sahu ID, McCarrick RM, Troxel KR, Zhang R, Smith HJ, Dunagan MM, Swartz MS, Rajan PV, Kroncke BM, Sanders CR, Lorigan GA (2013). DEER EPR measurements for membrane protein structures via bifunctional spin labels and lipodisq nanoparticles. Biochemistry.

[CR83] Sahu ID, Hustedt EJ, Ghimire H, Inbaraj JJ, McCarrick RM, Lorigan GA (2014). CW dipolar broadening EPR spectroscopy and mechanically aligned bilayers used to measure distance and relative orientation between two TOAC spin labels on an antimicrobial peptide. J Magn Reson.

[CR84] Sanders CR, Prosser RS (1998). Bicelles: a model membrane system for all seasons?. Structure.

[CR85] Sani MA, Separovic F (2015). Progression of NMR studies of membrane-active peptides from lipid bilayers to live cells. J Magn Reson.

[CR86] Schreier S, Bozelli JC, Marín N, Vieira RF, Nakaie CR (2012). The spin label amino acid TOAC and its uses in studies of peptides: chemical, physicochemical, spectroscopic, and conformational aspects. Biophys Rev.

[CR87] Seddon AM, Curnow P, Booth PJ (2004). Membrane proteins, lipids and detergents: not just a soap opera. Biochim et Biophys Acta (BBA) - Biomembr.

[CR88] Seebach D, Overhand M, Kühnle FNM, Martinoni B, Oberer L, Hommel U, Widmer H (1996). β-Peptides: Synthesis by Arndt-Eistert homologation with concomitant peptide coupling. Structure determination by NMR and CD spectroscopy and by X-ray crystallography Helical secondary structure of a β-hexapeptide in solution and its stability towards pepsin. Helvetica Chim Acta.

[CR89] Shen Y, Maupetit J, Derreumaux P, Tuffery P (2014). Improved PEP-FOLD approach for peptide and miniprotein structure prediction. J Chem Theory Comput.

[CR90] Shevelev GY, Krumkacheva OA, Lomzov AA, Kuzhelev AA, Rogozhnikova OY, Trukhin DV, Troitskaya TI, Tormyshev VM, Fedin MV, Pyshnyi DV, Bagryanskaya EG (2014). Physiological-temperature distance measurement in nucleic acid using triarylmethyl-based spin labels and pulsed dipolar EPR. Spectroscopy.

[CR91] Spindler PE, Schöps P, Bowen AM, Endeward B, Prisner T (2018) Shaped Pulses in EPR. In: Goldfarb D, Stoll S (eds) EPR Spectroscopy: Fundamentals and Methods. eMagRes Wiley and Sons 1477–1492. 10.1002/9780470034590.emrstm1520

[CR92] Srivastava M, Freed JH (2017). Singular value decomposition method to determine distance distributions in pulsed dipolar electron spin resonance. J Phys Chem Lett.

[CR93] Srivastava M, Freed JH (2019). Singular value decomposition method to determine distance distributions in pulsed dipolar electron spin resonance: II estimating uncertainty. J Phys Chem A.

[CR94] Stoller S, Sicoli G, Baranova TY, Bennati M, Diederichsen U (2011) TOPP: a novel nitroxide-labeled amino acid for epr distance measurements. Angew Chem Int Ed Engl 50:9743–9746. https://www.ncbi.nlm.nih.gov/pubmed/21898726 10.1002/anie.201103315 21898726

[CR95] Sun S, Neufeld CI, Latypov RF, Perez-Ramirez B, Xu Q (2015) Biophysical methods for the studies of protein-lipid/surfactant interactions. In: recent progress in colloid and surface chemistry with biological applications. ACS symposium series. American chemical society 1215 355–375. 10.1021/bk-2015-1215.ch017

[CR96] Taylor NMI, Manolaridis I, Jackson SM, Kowal J, Stahlberg H, Locher KP (2017). Structure of the human multidrug transporter ABCG2. Nature.

[CR97] Tkach I, Bennati M (2013) Dual-mode microwave resonator device and method of electron spin resonance measurement. Patents EP2486416B1, US9287606B2. https://patents.google.com/patent/EP2486416B1/en, https://patents.google.com/patent/US9287606B2/en

[CR98] Tkach I, Sicoli G, Höbartner C, Bennati M (2011). A dual-mode microwave resonator for double electron–electron spin resonance spectroscopy at W-band microwave frequencies. J Magn Reson.

[CR99] Tkach I, Pornsuwan S, Hobartner C, Wachowius F, Sigurdsson ST, Baranova TY, Diederichsen U, Sicoli G, Bennati M (2013). Orientation selection in distance measurements between nitroxide spin labels at 94 GHz EPR with variable dual frequency irradiation. Phys Chem Chem Phys.

[CR100] Tominaga M, Barbosa SR, Poletti EF, Zukerman-Schpector J, Marchetto R, Schreier S, Paiva AC, Nakaie CR (2001) Fmoc-POAC: [(9-fluorenylmethyloxycarbonyl)-2,2,5,5-tetramethylpyrrolidine-N-oxyl-3-amino-4-carboxylic acid]: a novel protected spin labeled beta-amino acid for peptide and protein chemistry. Chem Pharm Bull 49:1027–1029. 10.1248/cpb.49.1027 10.1248/cpb.49.1027 11515572

[CR101] Upadhyay AK, Borbat PP, Wang J, Freed JH, Edmondson DE (2008). Determination of the oligomeric states of human and rat monoamine oxidases in the outer mitochondrial membrane and octyl beta-D-glucopyranoside micelles using pulsed dipolar electron spin resonance spectroscopy. Biochemistry.

[CR102] Van Doorslaer S (2018) Hyperfine Spectroscopy: ESEEM. In: Goldfarb D, Stoll S (eds) EPR Spectroscopy: fundamentals and methods. eMagRes Wiley and Sons 51–70 10.1002/9780470034590.emrstm1517

[CR103] Wegner J, Valora G, Halbmair K, Kehl A, Worbs B, Bennati M, Diederichsen U (2019). Semi-rigid nitroxide spin label for long-range EPR distance measurements of lipid bilayer embedded beta-peptides. Chem-Eur J.

[CR104] Wirmer-Bartoschek J, Bartoschek S (2012). NMR in drug discovery on membrane proteins. Future Med Chem.

[CR105] Worswick SG, Spencer JA, Jeschke G, Kuprov I (2018) Deep neural network processing of DEER data Sci Adv 4(8):eaat5218. https://www.ncbi.nlm.nih.gov/pubmed/30151430 10.1126/sciadv.aat5218 PMC6108566 30151430

[CR106] Wright K, Sarciaux M, Wakselman M, Mazaleyrat JP, Crisma M, Formaggio F, Peggion C, Toffoletti A, Corvaja C, Toniolo C (2005) Synthesis of the spin-labelled beta-amino acids cis- and trans-beta-TOAC, and conformational study of a trans-beta-TOAC/ACHC hexapeptide. Biopolymers 80:574–574

[CR107] Wright K, Sarciaux M, Wakselman M, Mazaleyrat JP, Crisma M, Formaggio F, Peggion C, Toffoletti A, Corvaja C, Toniolo C (2006) Synthesis of the spin-labelled beta-amino acids cis- and trans-beta-TOAC, and a preliminary conformational study of trans-beta-TOACtrans-ACHC peptides. Understand Biol Using Pept. 10.1007/978-0-387-26575-9_241

[CR108] Wright K, Sarciaux M, de Castries A, Wakselman M, Mazaleyrat JP, Toffoletti A, Corvaja C, Crisma M, Peggion C, Formaggio F, Toniolo C (2007) Synthesis of enantiomerically pure cis- and trans-4-amino-l-oxyl-2,2,6,6-tetramethylpiperidine-3-carboxylic acid: a spin-labelled, cyclic, chiral beta-amino acid, and 3D-Structural analysis of a doubly spin-labelled beta-hexapeptide. Eur J Org Chem 2007:3133–3144. 10.1002/ejoc.200700153

[CR109] Yagi H, Banerjee D, Graham B, Huber T, Goldfarb D, Otting G (2011). Gadolinium tagging for high-precision measurements of 6 nm distances in protein assemblies by EPR. J Am Chem Soc.

[CR110] Zanker PP, Jeschke G, Goldfarb D (2005) Distance measurements between paramagnetic centers and a planar object by matrix Mims electron nuclear double resonance J Chem Phys 122:024515. https://aip.scitation.org/doi/pdf/10.1063/1.1828435 10.1063/1.1828435 15638606

[CR111] Zhang XJ, Cekan P, Sigurdsson ST, Qin PZ (2009). Studying Rna using site-directed spin-labeling and continuous-wave electron paramagnetic resonance spectroscopy. Method Enzymol.

